# Label free identification of the different status of anemia disease using optimized double-slot cascaded microring resonator

**DOI:** 10.1038/s41598-022-09504-2

**Published:** 2022-04-01

**Authors:** Mahdi Bahadoran, Abbas Kalate Seyfari, Parisa Sanati, Lee Suan Chua

**Affiliations:** 1 Department of Physics, Shiraz University of Technology, 31371555 Shiraz, Fars Iran; 2 Burn and Wound Healing Research Centre, Shiraz University of Medical Sciences, 71345-1978 Shiraz, Iran; 3 Institute of Bioproduct Development, Universiti Teknologi Malaysia, 81310 UTM Skudai, Johor Malaysia; 4 Department of Bioprocess and Polymer Engineering, School of Chemical and Energy Engineering, Faculty of Engineering, Universiti Teknologi Malaysia, 81310 UTM Skudai, Johor Malaysia

**Keywords:** Optical techniques, Micro-optics

## Abstract

An optical-based label-free biosensors including two indirectly coupled double-slot-waveguide-based microring resonator was designed and optimized for sensing purpose. Then, the optimized system was applied for the detection of hemoglobin concentration in anemia disease. The results were simulated based on the variational finite-difference time domain (varFDTD) method using the Lumerical software (Mode solutions) and the optimum geometrical parameters were determined to realize an optimum light transmission via the sensor. Nine different concentrations of hemoglobin in men and women were applied into the sensor and the status of anemia was identified based on the patients’ gender and different status of anemia disease, including the normal, mild, moderate, severe and life-threatening status. A sensitivity as high as 1024 nm/RIU with the minimum deflection limit of 4.88 × 10^–6^ RIU were measured for this biosensor, which introduces a high precision and micro-scale lab-on-a-chip micro device for health monitoring of the anemia.

## Introduction

Design of microstructural and low price sensor for a fast detection of hemoglobin concentration of human whole blood is significant for the detection of anemia status. Anemia arises when the red blood cells unable to carry enough oxygen to the cells and tissues. Based on the blood hemoglobin level, anemia typically divided into four states, including: mild, moderate, severe and life threatening status^1^. Anemia generally arises several disorders like overall weakness, change of skin color, short breath, dizziness, raising cardiac output and heart failure that may lead to death^2^. According to the World Health Organization, almost a quarter of world population (1.62 billion people) suffered from anemia during 1993–2005^3^. Anemia is also a key biomarker for several diseases like polychythemiavera^4^, cardiovascular^5^and lung cancer^6^. There exist several clinical laboratory measurement methods for diagnosis of anemia, such as the complete blood count (CBC)^7^, Sodium Lauryl Sulfate (SLS)^8^, the hemiglobincyanide-spectrophotometric^9^ and Azide-methemoglobin^10^. All of these clinical based methods are invasive approach that suffer from time-consuming process of lab analyzing.

The label-free detection (LFD) is a highly sensitive approach in biosensors that provides a straightforward and natural detection of biological samples, free from implanting any fluorescence dyes, markers or impurities into bio-cells^11^. The optical-based label-free biosensors have been attracting great attention in recent years due to their exclusive features like high sensitivity, convenient price, miniature size and immunity to environmental condition like humidity, spark and electromagnetic (EM) interference. Due to these characteristics, these biosensors have found lots of interests in bacteria detection^12–14^, drug screening^15^ detection of different cancers^11,16^ and quantitative monitoring of body performance^17^. The increasing tendency in the optical based biosensors is to design a miniaturized, high sensitive and cost effective sensor that provides fully-automated biological analysis and clinical diagnostics on a chip that known as lab-on-a-chip devices^18^. The key principle in optical biosensors is the light interaction with the bio-cells that lead to a particular spectral shift in the output light^19^.

Aside from the clinical laboratory methods, several optical methods have been introduced for the detection of hemoglobin concentration. Graphene based chalcogenide fiber applied for hemoglobin detection in^20^ that As40S60 used as a fiber core and a part of the cladding was stripped off and coated with a layer of chalcogenide glass, then this part dipped in the bio-sample. A detection limit of 18 μg/dL and sensitivity of 6.71 × 10 − 4 per g/dL was reported over the wavelength of 1000 nm. In another approach, the photonics crystal fibers have been used for hemoglobin detection. A silicon-based photonic crystal with a micro-size sensing cavity used as a biosensor and a sensitivity of 272.43 nm/RIU with quality factor of 3000 was measured in^21^. Nano-cavities implemented on a silica-based photonic crystal fiber and form nano ring resonator then applied for detection of glycated hemoglobin that reported a sensitivity of 690 nm/RIU with a quality factor of 500^22^. In the same approach, the silicon rods were located in the slab of silica and designed a photonic crystal based micro ring resonator for detection of hemoglobin concentrations based on the age and the gender of patients. The results then extended for identifying different types of thalassemia. This sensor operating range was over the wavelength of 1386–1419 nm and the sensitivity of 700 nm/RIU with a quality factor of 2700 were reporte^23^. Porous silicon based side-wall Bragg-grating resonator on the substrate of silica was used for sensing of various bio-cells, including blood plasma and hemoglobin that measured a sensitivity of 387.48 nm/RIU^24^. Plasmonics-based sensor consisted of two nano-size elliptical resonator etched on a gold plate was used for detection of hemoglobin concentration in^25^. This kind of sensors work based on the reflection of the electromagnetic wave from the sensor and measured a high sensitivity of 1100 nm/RIU with a figure of merit as high as 224 RIU^-1^. Microring resonator-based biosensors have also been used for detection of biological samples especially hemoglobin. An all-pass ring resonator from silica was applied as whispering gallery mode biosensor and a sensitivity of 361.3 nm/RIU with a Q-factor of 1143 were achieved for the resonance peaks between 1470 and1480 nm^26^. The superiority of microring based sensors^28–32^ rather than other optical based sensors are in achieving a sensor with high efficiency, low power consumption, convenient cost, high Q-factor and compatibility of fabrication process with conventional CMOS technology.

In this work, a system of two indirectly coupled double-slot-waveguide-based microring resonator was optimized and suggested for the detection of hemoglobin concentration in anemia disease. The sensor layout was optimized by considering the geometrical parameters of the ring’s waveguide to achieve an optimum light transmission through the system. The results were simulated using variational finite-difference time domain (varFDTD) method. The optimized sensor was applied to classify the patients suffering from anemia based on their gender and various anemia status from normal to life-threatening phase. Our proposed micro-size sensor takes advantage of high sensitivity, high precision, and quick detection of anemia status, moreover it can be used potentially as a practical optical sensor for individual health monitoring.

## Sensor structure

**Figure 1:**
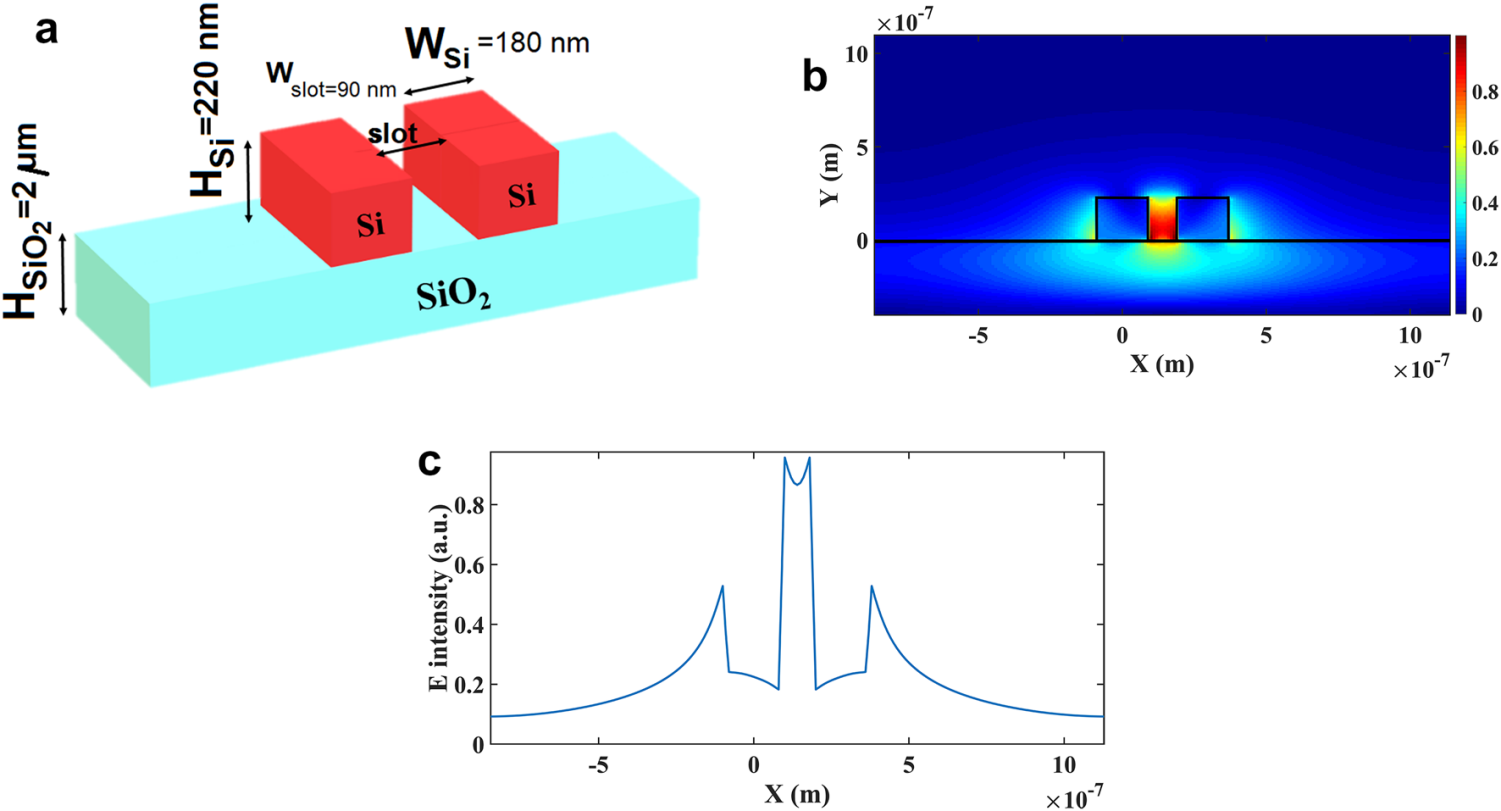
(**a**) The layout of SOI slot bus-waveguide (**b**) the TE mode profiles of the single slot bus-waveguide, (**c**) the distribution of electric field intensity via the single slot bus-waveguide.

Applying a slot in a common bus-waveguide can confine the quasi-TE mode in the slot region. As illustrated in Fig. 1-a, the applied single slot bus-waveguide consisted of two silicon ribs with the height and width of 220 nm and 180 nm, respectively, which placed on a silica substrate with a thickness of 2 μm. Theoretically, the slot region can enhance the light-matter interaction and potentially improves the sensitivity of the sensor. The width of slot shall be adjusted precisely since applying too wide slot may weaken the electrical strength through the slot region while selecting too narrow slot prevents entering of biological molecules into the slot area. Here, the width of slot region is selected to be 90 nm.

**Figure 2:**
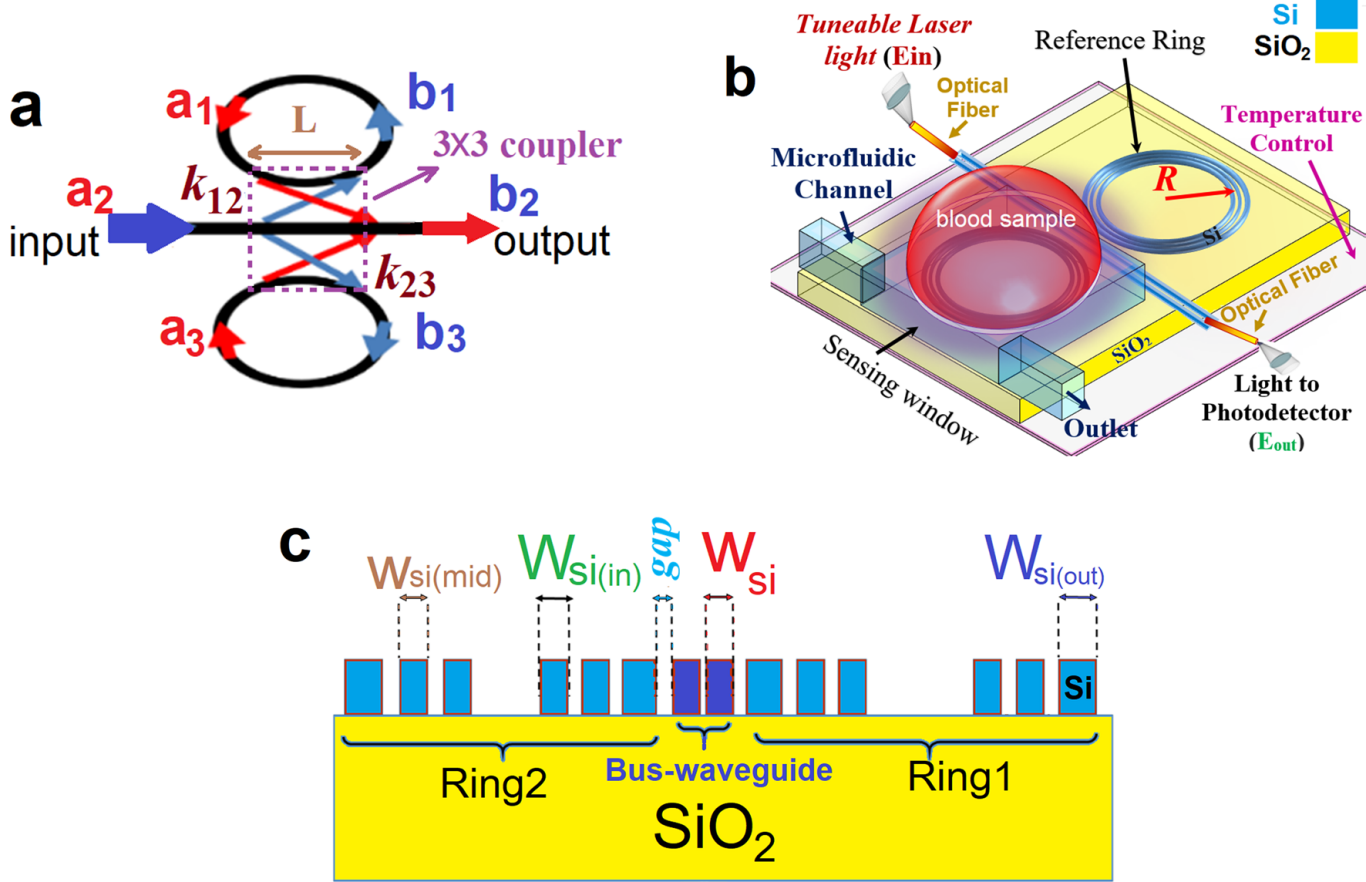
(**a**) The waveguide layout of an indirectly coupled cascaded ring resonator (ICCRR) comprised of a linearly distributed 3 × 3 coupler (**b**) a 3D structure of ICCRR sensor (**c**) the waveguide cross section of ICCRR sensor.

Realizing a light confinement in the slot region is one of the requirements of sensing application. As demonstrated in Fig. 1b,c, a high confinement of TE mode and an intensified electric field were realized within the slot region for the applied geometry in bus-waveguide. The double slot waveguide was used for the indirectly coupled cascaded ring resonators (ICCRR) as shown in Fig. 2. A single slot bus-waveguide was placed between two double-slot microring resonators that both microrings have the same radius of 5.0 μm. In practice, one ring can be used as the sensing ring and the other as the reference. The test sample (analyte or gas) can be pumped into the sensing window via the microfluidic channel. In order to achieve a high confinement of TE mode in the slot area and improve the efficiency of the sensor, an asymmetric double-slot waveguide was applied for the rings waveguides. An input light from a tunable laser source fed into the single slot bus-waveguide and light coupled evanescently into both rings via the 3 × 3 coupler. After satisfying the resonance condition of each ring, a fraction of propagating light through each ring will be re-coupled into the bus-waveguide and guided into the output port. A cascaded resonator comprised of a 3 × 3 coupler is demonstrated in Fig. 2-a. The coupler consisted of three waveguides (a bus-waveguide and two ring resonator waveguides) that are linearly distributed in the coupling region. Three input optical signals (a1, a2 and a3) were coupled into the coupling region and experiencing a clockwise rotation through the up-side ring and a counter-clockwise rotation via the down-side ring. The light fulfills the resonance condition of each ring while propagating via the rings then rejoin the coupling region and exit via three output paths of b1, b2 and b3. Based on the coupled mode theory, the optical transfer function of indirectly coupled cascaded ring resonator (ICCRR) was given by^27^

1 $$ OTF = \left| {\frac{Eout}{{Ein}}} \right| = \left| {\frac{{\beta 2 - \rho 1\left( { - \sigma 2\beta 1 + \sigma 1\beta 2} \right) - \rho 3\left( { - \gamma 2\beta 3 + \gamma 3\beta 2) + \rho 1\rho 3} \right)}}{{1 - \gamma 2\rho 3 - \sigma 1\rho 1 + \rho 1\rho 3\left( {\sigma 1\gamma 3\sigma - \sigma 3\gamma 1} \right)}}} \right| $$

where $$ \rho = {\text{exp}}\left( { - 0.5\alpha L_{n} } \right){\text{exp}}\left( { - ikn_{g} L_{n} } \right)$$ represents the light transmission parameter^28,29^, α indicates the internal loss factor of waveguide, $$L_{n}$$ is the length of each ring, $$n_{g} $$ shows the group index of the waveguide and $$k$$ represents the vacuum wave number. The $$ \beta_{n} ,\sigma_{n}$$ and $$\gamma_{n} $$(n = 1,2,3) in Eq. (1) were defined as follows^30^

2 $$ \left( {\begin{array}{*{20}c} {\sigma 1} & {\beta 1} & {\gamma 1} \\ {\sigma 2} & {\beta 2} & {\gamma 2} \\ {\sigma 3} & {\beta 3} & {\gamma 3} \\ \end{array} } \right) = \frac{1}{p}\left( {\begin{array}{*{20}c} {k_{12}^{2} \cos \varphi + k_{23}^{2} } & {ik_{12} \sin \varphi } & {k_{12} k_{23} (\cos \varphi + 1)} \\ {i\sqrt {pk_{12} \sin \varphi } } & {p\cos \varphi } & {i\sqrt {pk_{12} \sin \varphi } } \\ {k_{23}^{2} k_{12} (\cos \varphi + 1)} & {ik_{12} \sin \varphi } & {k_{23}^{2} \cos \varphi + k_{12}^{2} } \\ \end{array} } \right) $$

here $$, \varphi = l\sqrt p$$ , that $$l$$ displays the length of 3 × 3 coupling region, and $$p = k_{12}^{2} + k_{23}^{2}$$. The coupling strength between upside and downside rings shown by $$ k_{12}$$ and $$k_{23}$$, respectively. Equation (2) represented the optical transfer matrix for a coupler with 3 inputs and 3 outputs (3 × 3 coupler) that has an interaction coupling length of L (see Fig. 2a).

## Optimization of ICCRR sensor

The structure of the ICCRR sensor is illustrated in Fig. 2-b. The sensor formed by a single slot silicon bus-waveguide that coupled into two silicon microring resonators via a 3 × 3 coupler. A silica surface with the thickness of 2.0 µm was used as the substrate of the system. Both rings have the same radii and formed by the double-slot waveguides with a height of 220 nm. In this step, all the gaps and slots were selected to be 100 nm. A beam of light, Ein, from a tunable laser with the center wavelength of 1525 nm was fed into the straight bus-waveguide and coupled into the both rings by means of the 3 × 3 coupler. The light behavior through the ICCRR sensor was simulated using the varFDTD method, which is known as 2.5 dimensional FDTD. The varFDTD solver works by collapsing a 3D geometry to 2D set of effective indices that can be solved with 2D FDTD. The varFDTD can provide results comparable to 3D FDTD using only the simulation time and memory of a 2D FDTD simulation that is much faster and uses less memory compared to a 3D FDTD simulation. Since the varFDTD solver takes advantage of Hammer and Ivanova approach^31^, it can accurately describe light propagation in integrated optical systems that support two vertical modes with different polarizations like planar and ridge waveguide-based systems and photonic crystals.

Archiving a high sensitivity and high quality factor are desirable in designing of optical-based sensors. Change in the geometry of the sensor layout can change the interaction of the evanescent light with the bio-cells via the sensing ring that consequently can modify a spectral shift in the output light. The quality factor is defined as^32^

3 $$ Q - factor = \frac{{{\uplambda }_{{\text{R}}} }}{{{\text{FWHM}}_{{\text{R}}} }} $$

That ${\uplambda }_{{\text{R}}}$ and ${\text{FWHM}}_{{\text{R}}}$ represent the resonance wavelength and full-width-at-half-maximum (FWHM) of resonance peak, respectively. The locus of resonance peaks and the FWHM of each resonance peak correlate to the geometrical features of the layout such as the circumference of each ring, waveguide width and height, width and depth of the slots and the coupling gaps. Hence, in addition to the material of the optical path (waveguide), the geometry of the sensor is an effective factor in improving the sensitivity and Q-factor. Here, we took into account some geometric factors of the ICCRR sensor to reach the optimum structure. The considered geometrical factors are as follows: the length of straight section in rings (${\text{Lc}}$), the minimum spacing gap between the bus-waveguide and the waveguide of each ring (shown by “gap” in Fig. 2-c), the width of the rib between the slots (${\text{WSi\_mid}}$), the width of lateral ribs (${\text{WSi\_out}}$ and ${\text{WSi\_in}}$) and a parameter for evaluating the waveguide asymmetry (AP).

**Figure 3:**
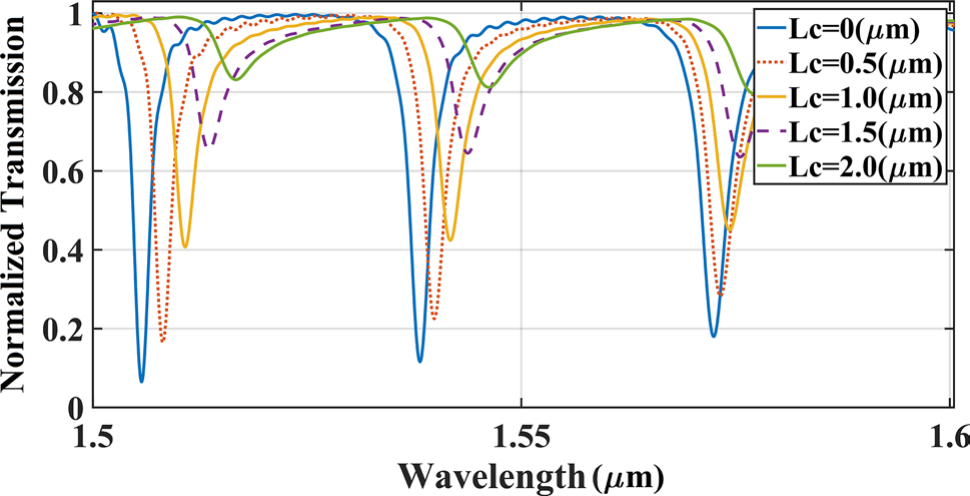
The transmission spectrum of ICCRR versus different 3 × 3 coupling length of ${\text{Lc}}$.

In the first step, the best length of ${\text{Lc}}$ was determined to achieve an output signal with high amplitude and narrow resonance peaks. This length was varied from 0 to 2.0 µm by step size of 0.5 µm and the light transmission versus wavelength were simulated using the varFDTD method as shown in Fig. 3. Results show that a larger coupling length contributes to generate a wider output signal with a smaller amplitude. Here, the maximum output power belongs to circular rings with the ${\text{Lc}}$ equal to 0 µm.

**Figure 4:**
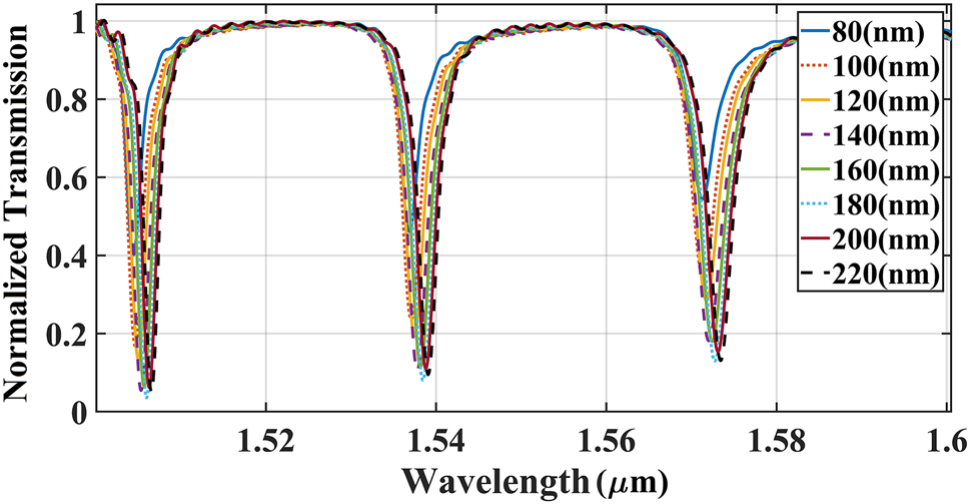
Effect of coupling size of the gap in the light transmission spectrum via ICCRR sensor for ${\text{Lc = 0}}$ .

The second step was to measure the ideal gap space between the rings and bus-waveguide. The transmission spectrum of ICCRR in terms of gap size is depicted in Fig. 4. The coupling gap was altered from a minimum possible gap value of 80 nm, to 220 nm with a step size of 20 nm. The output power amplitude was increased by extending the space from 80 to 180 nm. Nevertheless, applying the gaps larger than 180 nm lead to the smaller output power. Thus, the ideal gap in this step was selected to be180 nm.

**Figure 5:**
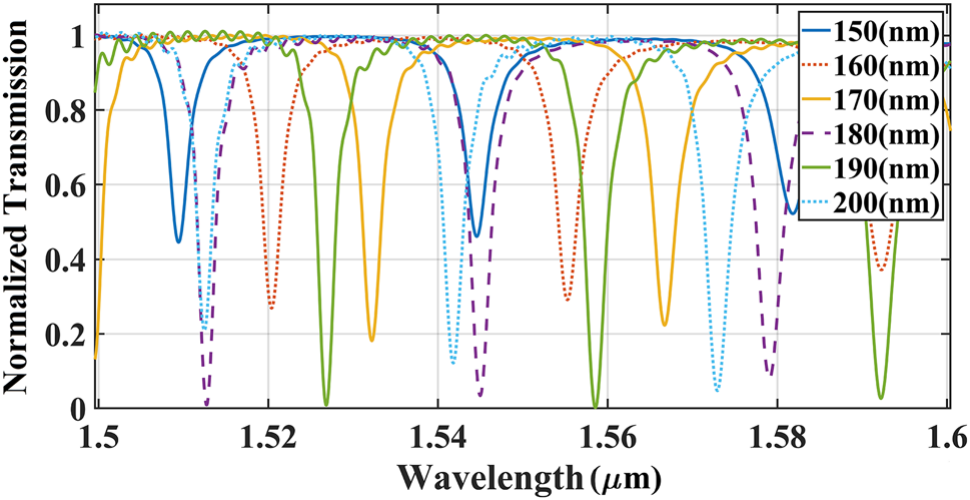
The transmission spectrum of ICCRR in terms of width of the outer ring ${\text{WSi\_out}}$. The circular rings with a gap size of 180 nm used for simulation.

As illustrated in the Fig. 2c, the double-slot ring resonator consisted of three concentric rings, including outer, middle and inner rings. In this step, we focused on determining of the ideal width for the outer ring (${\text{WSi\_out}}$). To this end, the width of the outer ring (outer rib) was altered from 150 to 200 nm and the transmission spectrum for each case was simulated as shown in Fig. 5. An uptrend was realized in the output resonance peaks of ICCRR while the ${\text{WSi\_out}}$ was enlarged from 150 to 190 nm, however applying the outer rib wider than190 nm caused a decrease in the output power. Therefore, the width of 190 nm has been chosen for ${\text{WSi\_out}}$ width.

**Figure 6:**
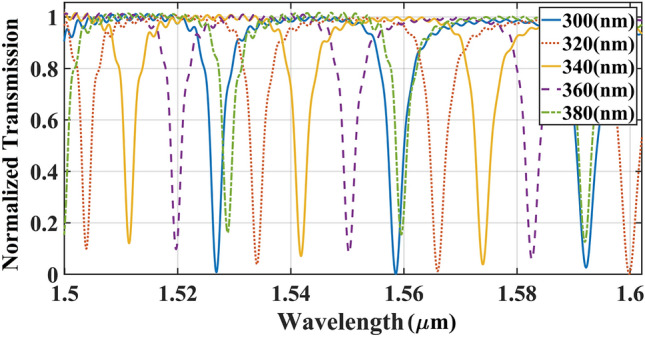
Effect of the total middle and inner rib widths of double-slot ring resonator waveguide on the light transmission spectrum of ICCRR system. Results simulated based on the fixed parameters of ${\text{Lc = 0}}$ $\mu$ m, ${\text{gap = 180 nm}}$, and ${\text{WSi\_out = 190 nm}}$.

Before measuring the middle and inner rib widths, their total ideal width (${\text{WSi\_sum = WSi\_in + WSi\_mid}}$ ) was calculated as the fourth step. The calculated parameters in the last steps were used as the fixed parameters in the simulation $(Lc, gap, {\text{WSi\_out) = (0, 180 nm, 190 nm)}}$, then the total width excluding the slot was modified from 300 nm and extended to 380 nm with an increment step of 20 nm. As shown in Fig. 6, an increase of total width from 300 to 380 nm, gives rise to a moderate decrease in the output transmission, hence the suitable size for total middle and inner rings (${\text{WSi\_out}}$) is 300 nm.

**Figure 7:**
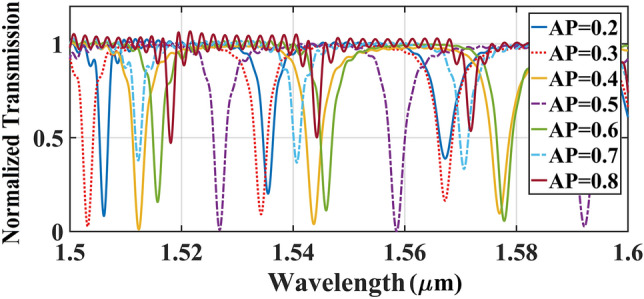
The transmission spectrum of ICCRR versus the asymmetry parameter (AP) for the applied parameters of ${\text{Lc = 0}}$ µm, ${\text{gap = 180 nm}}$, ${\text{WSi\_out = 190 nm}}$ and ${\text{WSi\_sum = 300 nm}}$.

To determine the position of the slots in the ring waveguide, an asymmetric parameter (AP) was defined as the ratio of the middle rib to the total ideal width ($${\text{AP = WSi\_mid/WSi\_sum}}$$ ).The transmission spectrum for the variation of AP from 0.2 to 0.8 were simulated by adjusting the total width on the fixed value of 300 nm. As shown in Fig. 7, the output peaks get increased gradually by increasing the AP from 0.2 to 0.5, and for AP values higher than 0.5 the power amplitude was reduced. As a result, the optimum value of AP was selected to be 0.5 that means both middle and inner ribs of ring waveguide must have the same widths equal to $${\text{WSi\_mid = WSi\_in = 150 nm}}$$. Hence, an asymmetric double-slot ring waveguide for optimum light transition will have the following widths $${\text{(WSi\_in}}$$ , $${\text{WSi\_mid}}$$ ,$${\text{WSi\_out) = }}$$$${\text{(150, 150, 190) nm}}{.}$$.

**Figure 8:**
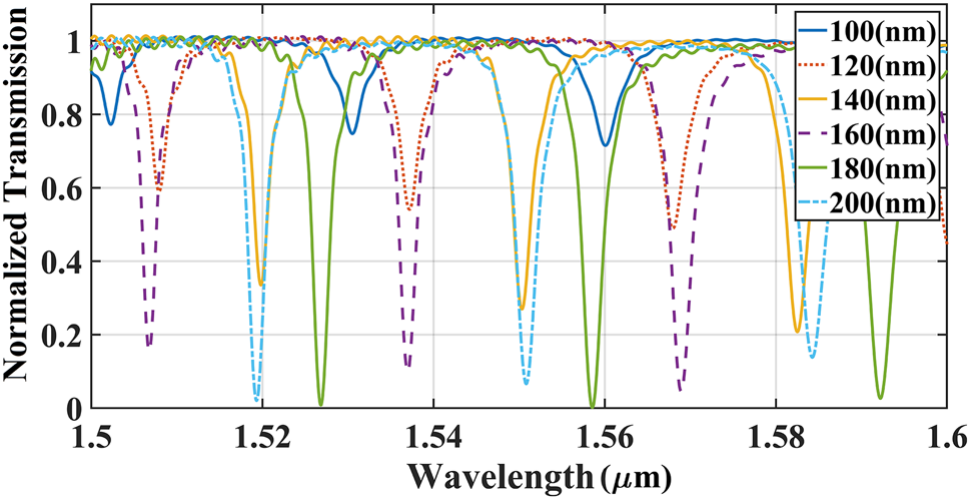
The effect of equal slot widths of the ring waveguide in the transmission spectrum considering the fixed parameters of ${\text{Lc = 0}}$ µm, ${\text{gap = 180 nm}}$, ${\text{WSi\_out = 190 nm, WSi\_mid = WSi\_in = 150 nm}}$ nm.

The width of the slots in the ring double-slot waveguide was calculated in the last step. The transmission spectrum in terms of different width of the inner ring slots is delineated in Fig. 8. The width of both slots was changed from 100 to 200 nm. An uptrend in the amplitudes was realized for extending the width from 100 to 180 nm and the maximum power was realized in the slot width of 180 nm, however applying the broaden slots higher than 180 nm brings about low quality signals. Consequently, the optimum value of 180 nm was opted for both slot widths inside the ring’s waveguide. The optimum structural parameters for double-slot waveguide-based indirectly coupled cascaded ring resonator that realized by varFDTD method are given in Table 1.

**Table 1:** The achieved optimum parameters for ICCRR sensor.

Parameters	Description	Optimum value
Slot-BWG	Slot width in the bus-waveguide	90 nm
WSi-BWG	Width of each rib of Silicon bus-waveguide	180 nm
L-BWG	Length of bus waveguide	25 μm
R	Microring resonators radius	5 μm
${\text{Lc}}$	Length of straight section in rings	0 μm
L	Interaction coupling length between bus-waveguide and rings	500 nm
gap	The space between bus waveguide and rings	180 nm
Slot-ring	The length between the ribs inside the waveguide	180 nm
H_Si_	Height of all Silicon ribs	220 nm
H_SiO2_	Height of all Silica substrate	2 μm
WSi_out	Width of outer Silicon ring	190 nm
WSi_mid	Width of middle Silicon ring	150 nm
WSi_in	Width of inner Silicon ring	150 nm
AP	Asymmetry parameter	0.5
Wslot1	Width of outer slot of rings	180 nm
Wslot2	Width of inner slot of rings	180 m
total device size	Width × Length × Height	12 μm × 24 μm × 2.22 μm

**Figure 9:**
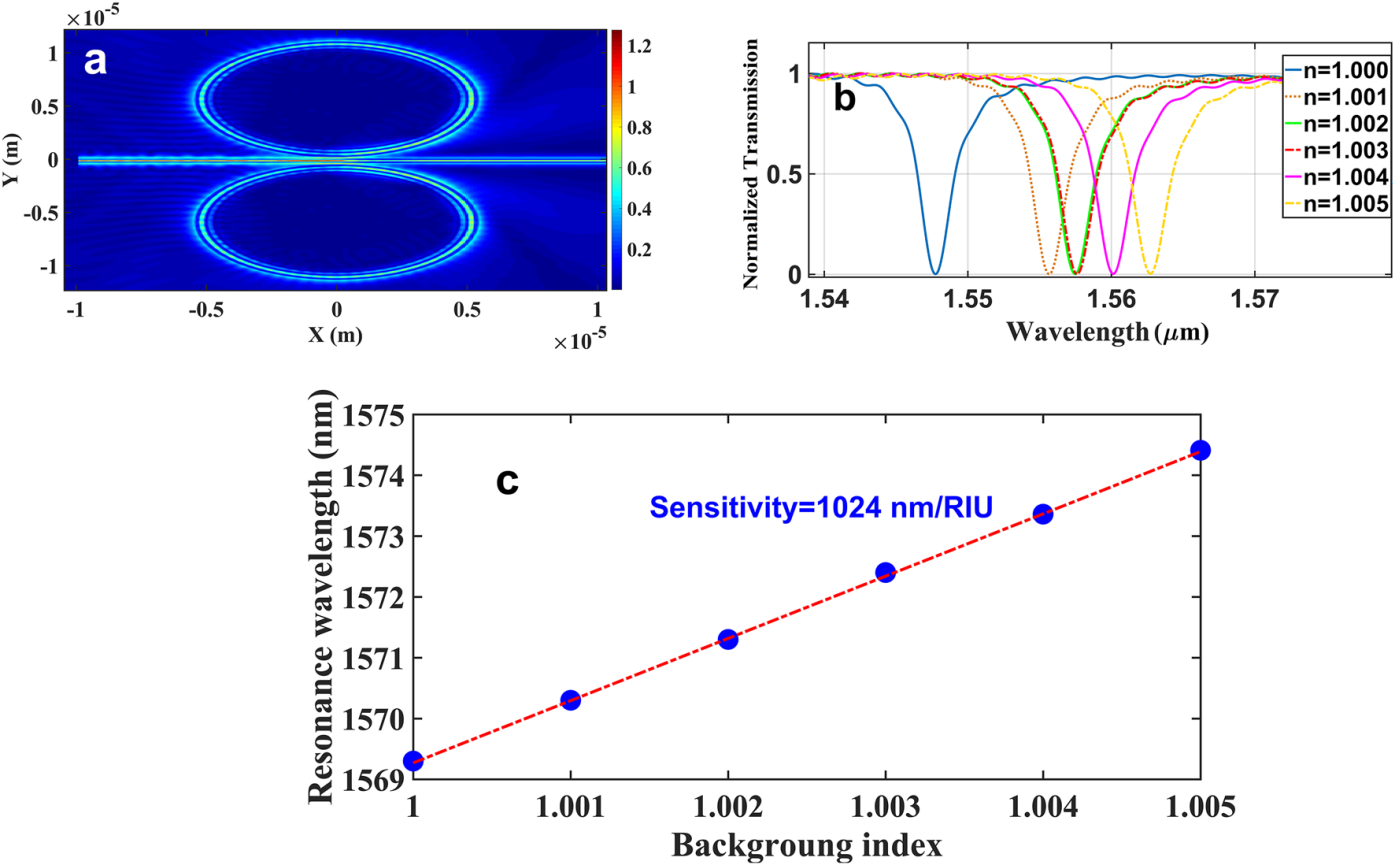
(**a**) The electric field mode profile propagating via the ICCRR system. (**b**) The wavelength shift in resonance peaks for different background indices (**c**) sensing line for ICCRR system.

Applying these structural parameters to the ICCRR system gives rise to detect resonances with a FWHM as narrows as 2.83mn that leads to quality factor of 570 (Eq. (3)) and realizing a free spectral range as large as 33.6 nm. The electric field mode profile propagating through ICCRR system is shown in Fig. 9a. The mode is confined in the slot areas, which provides the enhancement of the light interaction with the test sample in slots. It makes the system as an appropriate candidate for sensing applications. In order to check the sensing functionality of the device, the ICCRR sensor put under the various background refractive indices from n = 1.000 to 1.005 with step size of 0.001 and the spectral response of output light was measured.

The results presented in Fig. 9b, and the details of each resonance peak are given in Table2. In the microring-based devices, the sensitivity, quality factor and figure of merit (FOM) are calculated by Δλ_peak_ /Δn, λ_peak_/FWHM and sensitivity/FWHM ,respectively^33,34^, which Δλ_peak_ represents the spectral shift of the resonance peaks, Δn shows the variation in the index of the test sample and FWHM shows the full width ant half maximum. As shown in Table 2, the maximum sensitivity of 1033 nm/RIU with FOM of 366.6 and quality factor of 524.1 can be realized by the ICCRR sensor. The resonance wavelength shift in terms of variations of the background refractive index simulated in Fig. 9-c that form the sensing line for the system. According to this line, the sensitivity of ICCRR sensor is 1024 nm/RIU and the minimum deflection limit corresponds to the archived sensitivity is 14.88 × 10^–6^ RIU.

**Table 2:** The characteristics of resonance peaks under the variation of background index.

Background index	Resonance Wavelength (nm)	Spectral shift	FWHM (nm)	Sensitivity (nm/RIU)	FOM (1/RIU)	Quality factor
1.000	1569.30	–	2.9	–	–	541.1
1.001	1570.30	1.00	3.0	1000	333.3	523.4
1.002	1571.30	2.00	3.0	1000	333.3	523.7
1.003	1572.40	3.10	3.0	1033	366.6	524.1
1.004	1573.36	4.06	3.1	1015	290.3	507.5
1.005	1574.41	5.11	3.0	1022	333.3	524.8

## Applying the ICCRR sensor for detection of hemoglobin concentration

**Figure 10:**
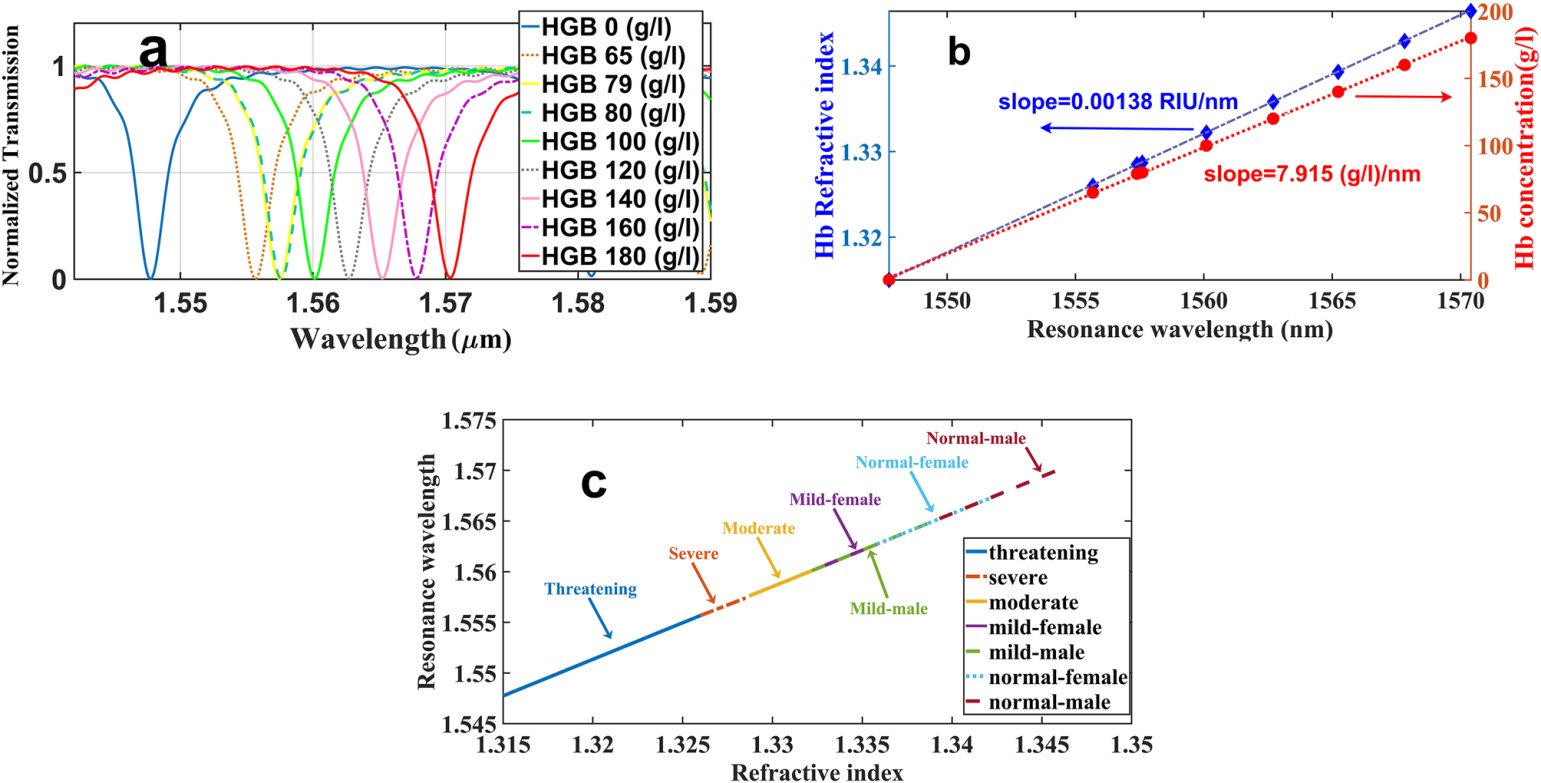
(**a**) The resonance wavelength shift in terms of variations of analyte hemoglobin concentration (**b**) the hemoglobin (Hb) refractive index (blue) and Hb concentration (red) sensing lines against the wavelength of the resonance peaks (**c**) the sensing line for the diagnosis of anemia patients’ status.

The anemia diagnosis status depends on the hemoglobin concentration in the human whole blood. The refractive indices of different hemoglobin solutions (obtained from human whole blood) have been measured using different techniques such as the multi wavelength Abbe refractometer^35^, Fresnel formula and spectral method^36^ and the spectroscopic phase microscopy^37^. The Sellmeier coefficients have been calculated for the specific concentrations of hemoglobin based on the experimental data^35^. The normal concentration of hemoglobin in men and women was measured to be in the range of 140–180 (g/l) and 120–160 (g/l), respectively. The patients with the mild status typically have the hemoglobin concentration of 100 (g/l) to the normal limit, while the concentration range of 80–100 (g/l) represents the moderate status. When the hemoglobin concentrations goes down between 65 and 79 (g/l), the patients are classified in the severe status and realizing the hemoglobin concentrations less than 65 (g/l) denotes for life-threatening phase^38^. We applied these nine values of hemoglobin concentration to find a sensing line for the diagnosis of the status of anemias disease. The refractive index of different concentration of hemoglobin in the range of 0–250 (g/l) were experimentally calculated in terms of wavelength in ^35^. According to the Abbe refractometer technique, the refractive indices of 1.315, 1.3260395, 1.3285248, 1.3287024, 1.3322520, 1.3358036, 1.3393542, 1.3429048 and 1.3464554 correspond to the hemoglobin solutions with concentrations of 0 g/l, 65 g/l, 79 g/l, 80 g/l, 100 g/l, 120 g/l, 140 g/l, 160 g/l and 180 g/l, respectively. These data were measured in the wavelength of 1550 nm and room temperature of 23 °C^35^. Nine different hemoglobin concentrations were applied on the sensing window of the ICCRR sensor and the light transmission versus wavelength was monitored for each case. A sample with zero concentration is considered as base-line and the spectral shift of other samples were measured with respect to this base-line as shown in Fig. 10a. Two sensing lines based on the different concentrations of hemoglobin (red circles) as well as refractive indices were plotted versus resonance wavelengths in Fig. 10b. These lines are the key indicator for identifying the anemia different phases. A sensing line for the diagnosis of anemia patients’ status were plotted in Fig. 10c. Different phases of anemia disease are in a line with the same slope, but placed in the different areas. The line reveals that applying a sample from a life threatening patient (solid blue line) brings about realizing resonances in the wavelengths between 1547.74 and1555.68 nm. The sample took from anemia patient in severe phase can generate the resonances interval 1555.68 nm-1557.60 nm (brown dash-line), and the sample of patient suffering from moderate anemia can generate resonances in the range of 1557.60 nm–1560.10 nm (solid yellow line). The mild status of anemia started at 1560.1 nm, however, it limited based on the gender of the patients. The samples from women suffering from mild anemia lead to resonances between 1560.10 and 1562.70 nm, while those of taken from the men in the mild phase bring about realizing the resonances in 1560.10 nm–1565.23 nm. Results of normal people are also depends on the gender as resonances for normal men and normal women emerge in the spectral range of 1565.23 nm–1570.40 nm and 1562.70 nm–1567.82, respectively.

In comparison with the conventional optical based biosensor especially ring resonator based sensors given in Table 3, the realized sensitivity of the ICCRR sensor is considerably high, which is due to the optimized features of double-slot waveguide that provides a high interaction between light and bio-cells. Totally, this ICCRR sensor can facilitate and accelerate the diagnostic process of anemia patients. Moreover, the patients can continually monitor the phase of their anemia on a daily basis.

**Table 3:** Comparative sensitivity of different optical based sensors for detection of hemoglobin concentration. Here, DL, FOM and Q stand for the detection limit, a figure of merit and quality factor.

Sensor configuration	Sensitivity	Highlight	Simulation method	performance range	Ref
Graphene based chalcogenide fiber	6.71 × 10–4 per g/dL	DL = 18 μg/dL	intensity interrogation method	700 nm–1000 nm	^20^
One-dimensional photonic crystal based structure	323 nm/RIU (0.05 nm/(g/L)	FOM = 517 /RIU	transfer matrix method	1550 nm–1645 nm	^39^
Silicon-based photonic crystal nanocavity	272.43 nm/RIU	Q = 3000	FDTD and plane wave expansion	1560 nm–1680 nm	^21^
All-circular photonic crystal ring resonators	690 ± 50 nm/RIU	Q = 500 ± 500, FOM = 1400 ± 200 /RIU	FDTD	1530 nm–1580 nm	^22^
Photonic crystal based micro ring resonator	700 nm/RIU 2 nm/g/dL	Q = 2500 FOM = 200/RIU	FDTD	1386 nm –1419 nm	^23^
Tilted fiber gratings-based surface plasmon resonance	522.8 nm/RIU 8.144 nm/mg/mL	–	UV-inscription fabrication technique	1550 nm	^40^
Plasmonics-based refractive index sensor	1100 nm/RIU	FOM = 224 /RIU	FDTD	940 nm –1100 nm	^25^
WGM ring resonator	361.3 nm/RIU 6.1025 g/l for every 0.001 RI change	Q = 1143	FEM	1470 nm –1480 nm	^26^
two indirectly coupled double-slot-waveguide-based microring resonator	1024 nm/RIU 0.13 nm/(g/l)	FOM = 366.6 /RIU Q = 524.1 DL = 4.88 × 10^–6^	varFDTD	1530 nm–1610 nm	Present work

## Conclusions

An indirectly coupled cascaded double-slot-waveguide-based microring resonator was optimized then applied for detection of hemoglobin concentration in anemia disease. The optimum geometric dimension of the proposed sensor was determined to realize the optimum transmission of light through the sensor. The functionality of the sensor was checked for detection of different concentrations of hemoglobin in anemia patients and a sensing line was realized for the diagnosis of different phases of anemia disease. The results simulated based on varFDTD and a sensitivity as high as 1024 nm/RIU correspond to 0.13 nm/(g/l) was obtained. This proposed sensor can fulfill the expectations of achieving a low cost and high sensitive lab-on-a-chip device for monitoring of anemia status.

## Data Availability

The datasets used and/or analyzed during the current study available from the corresponding author on reasonable request.
